# Oxytocin in Huntington’s disease and the spectrum of amyotrophic lateral sclerosis-frontotemporal dementia

**DOI:** 10.3389/fnmol.2022.984317

**Published:** 2022-09-14

**Authors:** Sofia Bergh, Rachel Y. Cheong, Åsa Petersén, Sanaz Gabery

**Affiliations:** Translational Neuroendocrine Research Unit, Department of Experimental Medical Science, Lund University, Lund, Sweden

**Keywords:** oxytocin, Huntington’s disease, amyotrophic lateral sclerosis, frontotemporal dementia, neurodegenerative diseases, huntingtin, TDP-43

## Abstract

Neurodegenerative disorders (NDDs) such as Huntington’s disease (HD) and the spectrum of amyotrophic lateral sclerosis (ALS) and frontotemporal dementia (FTD) are characterized by progressive loss of selectively vulnerable populations of neurons. Although often associated with motor impairments, these NDDs share several commonalities in early symptoms and signs that extend beyond motor dysfunction. These include impairments in social cognition and psychiatric symptoms. Oxytocin (OXT) is a neuropeptide known to play a pivotal role in the regulation of social cognition as well as in emotional behaviors such as anxiety and depression. Here, we present an overview of key results implicating OXT in the pathology of HD, ALS and FTD and seek to identify commonalities across these NDDs. OXT is produced in the hypothalamus, a region in the brain that during the past decade has been shown to be affected in HD, ALS, and FTD. Several studies using human post-mortem neuropathological analyses, measurements of cerebrospinal fluid, experimental treatments with OXT as well as genetic animal models have collectively implicated an important role of central OXT in the development of altered social cognition and psychiatric features across these diseases. Understanding central OXT signaling may unveil the underlying mechanisms of early signs of the social cognitive impairment and the psychiatric features in NDDs. It is therefore possible that OXT might have potential therapeutic value for early disease intervention and better symptomatic treatment in NDDs.

## Introduction

Neurodegenerative disorders (NDDs) are a group of diseases caused by progressive and irreversible deterioration of neurons within the central nervous system (CNS). Current treatment is only symptomatic but does not modify disease progression or reverse the neuronal dysfunction. These disorders are characterized by selective cellular vulnerability to the pathogenic process that often involve the accumulation of disease-associated proteins. Important research questions are centered on understanding the underlying mechanisms of selective vulnerability as well as what changes occur early and could be targeted for therapeutic disease-modifying interventions. Recent work has indicated interesting similarities between Huntington’s disease (HD) and the spectrum of amyotrophic lateral sclerosis (ALS) and frontotemporal dementia (FTD) (Vercruysse et al., 2018). The concept of an ALS and FTD disease spectrum continuum has emerged, largely from an overlap in pathological and genetic associations between the two conditions (Neumann et al., 2006; Renton et al., 2011; Strong et al., 2017). Although previous research has mainly focused on the well-known disturbances in motor function and accompanying neuropathology of movement-regulating neurons for HD and ALS, recent work show early manifestation of psychiatric symptoms and altered social cognition as well as selective vulnerability of hypothalamic neurons in both HD and the spectrum of ALS and FTD disorders (Vercruysse et al., 2018; Gabery et al., 2021). Understanding the underlying mechanisms of selective vulnerability of hypothalamic neurons and the link to early psychiatric symptoms in these disorders may open up for novel avenues for therapeutic interventions for these disorders.

Several genes have been identified in the familial forms of these NDDs. HD is caused by an expansion of the trinucleotide CAG within the *huntingtin* (*HTT*) gene (HDCRG, 1993), while there are several known genetic mutations in ALS such as *superoxide dismutase 1 (SOD1), chromosome 9 open reading frame 72 (C9ORF72)* and, *trans-activation responsive RNA-binding protein (TARBP)*. The latter two genes are also affected in FTD as well as *progranulin (GRN)* and *microtubule associated protein tau (MAPT)* (Taylor et al., 2016; Greaves and Rohrer, 2019). Also, CAG expansions within the *ataxin-2* have also been shown to be associated with ALS (Elden et al., 2010).

Cognitive and social behavioral alterations are key early features that are present in HD, ALS, and FTD (Craufurd et al., 2001; Phukan et al., 2007; Paulsen et al., 2008; Steenland et al., 2010; Ahmed et al., 2014; Bott et al., 2014; McCarter et al., 2016; Herzog-Krzywoszanska and Krzywoszanski, 2019; Blasco et al., 2020; Boentert, 2020; Singh and Agrawal, 2021). In particular, social cognitive impairment (e.g., processing of facial expression of emotions, theory of mind, and empathy) in NDDs has the potential to disrupt interpersonal relationships, thereby eliminating the benefits that social interactions may bring for patients suffering from these debilitating conditions (Christidi et al., 2018). The underlying biological mechanisms mediating these features in NDDs are not well understood. However, given that both altered social cognition as well as psychiatric features constitute a common denominator in the early phases across all three diseases, it is likely that that there could also be common pathologies in HD, ALS, and FTD.

Hypothalamic alterations can be observed in all three NDDs, including loss of different hypothalamic neurons (Gabery et al., 2010, 2015, 2021; Vercruysse et al., 2018; Ahmed et al., 2021c). The neuropeptide oxytocin (OXT) has long been known to play a pivotal role in the regulation of complex social cognition and behaviors, including prosocial behavior and pair-bonding (Heinrichs et al., 2009; Galbally et al., 2011; Odent, 2013) as well as in emotional behavior including anxiety and depression (Neumann and Landgraf, 2012; Jurek and Neumann, 2018; Onaka and Takayanagi, 2019; Yoon and Kim, 2020). OXT is synthesized in the paraventricular nucleus (PVN) and supraoptic nucleus of the hypothalamus (Gimpl and Fahrenholz, 2001). OXT binds to OXT-receptors (OXTR) that are located throughout the brain most prominently within the limbic structures (Jurek and Neumann, 2018). Interestingly, in a recent study, distinct OXTR expression patterns have been shown in psychiatric disorders as well as in metabolic regulation processes across development in humans (Rokicki et al., 2022). Furthermore, genetic variation of OXTR has also been shown to be associated with social impairment in HD (Saiz-Rodríguez et al., 2022). When exogenously administered, OXT facilitates social encounters, improved social cognitive outcomes as well as emotion recognition in healthy and clinical groups characterized by social deficits, such as autism and social anxiety disorder (Heinrichs et al., 2009; Keech et al., 2018).

Other hypothalamic specific neuropeptides that have been studied in NDDs include hypocretin (orexin) which has been shown to be reduced in both HD and ALS (Gabery et al., 2010, 2021) as well as in Parkinson’s disease (Fronczek et al., 2007). Like OXT, hypocretin is exclusively produced in the hypothalamus and is involved in the regulation of sleep, emotion, and metabolism (Tyree et al., 2018). Other hypothalamic specific neurons such as vasopressin has also been shown to be reduced in HD (Gabery et al., 2010), but not in ALS or FTD (Piguet et al., 2011; Gabery et al., 2021). Hypothalamic expression of neuropeptide Y (NPY), a neuropeptide involved mostly in appetite regulation has also been studied and is preserved in HD, ALS, and FTD (Gabery et al., 2010; Piguet et al., 2011).

Hence, several studies have indicated that the OXT system may be implicated across both HD and the spectrum of ALS/FTD and therefore constitute an interesting common denominator (Gabery et al., 2010, 2015, 2021; Jesso et al., 2011; Finger et al., 2015). Given the role OXT has on social cognition and emotional regulation, there may be a link between the pathology in the OXT system identified in these disorders and some of the early social cognitive and psychiatric features. OXT system may provide a common therapeutic target for disease intervention. Understanding similarities and differences of these disorders including of how the OXT system is affected may provide important information in that direction. This review therefore focuses on the current state of knowledge regarding changes in the OXT system in these particular NDDs and highlights the major results obtained so far in this emerging field.

### Clinical features and neuropathology of Huntington’s disease

The clinical diagnosis of HD is based on a positive gene test in combination with the manifestation of overt motor disturbances including chorea (exaggerated involuntary movements), rigidity and gait imbalance (Novak and Tabrizi, 2010; Ross et al., 2014). The onset of motor disturbances usually occurs in midlife (30–55 years of age) followed by 20 years of disease progression. Individuals with HD also experience a range of non-motor symptoms and signs. These include cognitive changes, such as executive dysfunction and altered social cognition, as well as psychiatric symptoms such as anxiety, depression, irritability, and apathy (Duff et al., 2007; Paulsen et al., 2008). The disease-causing mutation is an expansion of a CAG trinucleotide repeat in the *HTT* gene which encodes an extended polyglutamine (Q) of the HTT protein (HDCRG, 1993; Ross et al., 2014). Individuals carrying more than 36Q will develop HD, but there is reduced penetrance if the patient is carrying between 36 and 39Q (Duyao et al., 1993). Despite a major hallmark of HD pathology being the formation of intraneuronal aggregates of the mutant HTT protein, the role of these aggregates in the pathogenesis is not well understood (DiFiglia et al., 1997; Ross and Shoulson, 2009; Cisbani and Cicchetti, 2012). In HD, the most pronounced neuropathology is observed in the striatum of the basal ganglia and the cerebral cortex which are regions associated with motor function (Vonsattel et al., 1985). The site of pathology for psychiatric signs and symptoms as well as altered social cognition is still not established but may in part be explained by dysfunctional neural circuities and neuronal cell death in the hypothalamus. Hypothalamic alterations have been observed decades before onset of motor disturbances (Politis et al., 2008; Soneson et al., 2010; Cheong et al., 2019). Recent studies have aimed to increase the understanding of the non-motor features of HD and in particular the role of OXT in this paradigm. A summary of the main results for the role of OXT in HD pathology can be found in Table 1.

**TABLE 1:** Summary of oxytocin (OXT) results in Huntington’s disease (HD).

Tissue	HD stage	Analysis	Treatment		Results	References
**Clinical HD**		IHC, stereology		↓	Number of OXT neurons	Gabery et al., 2010; Gabery et al., 2015
		IHC		=	Number of OXT neurons	van Wamelen et al., 2012
		IHC, stereology		↑	Atrophic OXT neurons	Gabery et al., 2010
	Premanifest and manifest	EIA, ELISA, RIA		=	OXT plasma level	Unti et al., 2018; Fisher et al., 2021; Hellem et al., 2022
	Premanifest	ELISA, SDMT, VDT		↑	OXT plasma levels correlated with ↑ executive function	Fisher et al., 2021
	Premanifest and manifest	ELISA, PBA		↑	OXT plasma levels correlated with ↓ depressive-symptoms	Fisher et al., 2021
	Manifest	EIA, faux-pas		↑	OXT correlated with ↑ social cognition	Unti et al., 2018
	Premanifest	fMRI, emotional face matching task	Intranasal OXT adm.	↑	Ability to process disgust stimuli	Labuschagne et al., 2018
	Manifest	RIA		↓	OXT CSF levels	Hellem et al., 2022
		RIA, MMSE, MoCA, TASIT, EHt, RME		↓	OXT CSF levels correlated with ↑ cognitive impairment	Hellem et al., 2022
**HD190QG** **(Mouse)**		RT-PCR		↓	OXT mRNA	Kotliarova et al., 2005
		IHC		=	Number of OXT neurons	Kotliarova et al., 2005
**BACHD** **(Mouse)**		RIA		↓	OXT plasma level	Cheong et al., 2020
		RIA, EPM, FST, SIT		↓	OXT plasma levels with ↑ depressive-, anxiety-like and altered social behavior	Cheong et al., 2020
		FST	Intranasal OXT adm.	↓	Depressive-like symptoms	Cheong et al., 2020
		IHC, stereology		=	Number of OXT neurons	Soylu-Kucharz et al., 2016
		IHC, stereology	QA injection	=	Number of OXT neurons	Henningsen et al., 2021
**R6/2** **(MOUSE)**		RT-PCR		↓	OXT mRNA levels	Kotliarova et al., 2005
		IHC, stereology		↓	Number of OXT neurons	Henningsen et al., 2021
		IHC, stereology	QA injection	=	Number of OXT neurons	Henningsen et al., 2021
**AAV-MHTT** **(Mouse)**		RT-PCR	mHTT AAV vector injection	↓	OXT mRNA	Hult et al., 2011
**NP-3** **(Rat)**		OFT, EPM, FST	icv OXT injection	↓	Anxiety- and depressive-like symptoms	Khodagholi et al., 2022
		Western blot, ellman method	icv OXT injection	↑	OXTR, mGluR2, GSH levels	Khodagholi et al., 2022
		Western blot	icv OXT injection	↓	mGluR5 levels	Khodagholi et al., 2022

↓: decrease in symptomatic or abundance, ↑: increase in abundance, =: no change in neuronal population. adm, administration; AAV, adeno-associated virus; EHt, emotion hexagon test; EIA, enzyme inhibition assay; ELISA, enzyme-linked immunosorbent assay; EPM, elevated plus maze; FST, forced swim test; IHC, immunohistochemistry; icv, intracerebroventricular injection; mHTT, mutant huntingtin; MoCA, Montreal cognitive assessment; MMSE, mini mental state examination; OFT, open field test; QA, quinolinic acid; RIA, radioimmunoassay; RME, reading the mind of the eyes; RT-PCR, real-time polymerase chain reaction; PBA, problem behaviors assessment; SDMT, symbol digit modalities test; SIT, social interaction test; TASIT, the awareness of social interference test; VDT, verbal fluency test.

### Changes in the oxytocin system in Huntington’s disease

The first clinical study to investigate OXT pathology in clinical HD was conducted by Gabery et al. (2010). Immunohistochemically processed post-mortem brain tissue from HD patients of different Vonsattel grades (grades 2–4) revealed a selective 45% OXT neuronal loss as well as a reduced cell size of the remaining OXT neurons. The Vonsattel grading system is a five-step grading system (0–4) for neurodegeneration in HD focusing on the striatum, the most affected brain region (Vonsattel et al., 1985). Furthermore, a case report based on one HD patient with Vonsattel grade 0 showed the same low number of OXT-expressing neurons as late-stage HD patients with Vonsattel grade 2–4 (Gabery et al., 2015). The same patient had deceased before the onset of any motor signs and symptoms, however, had developed anxiety and sleep disturbances (Gabery et al., 2015). These results suggest that early changes in hypothalamic neuronal populations expressing emotion-regulating neuropeptides could contribute to the early behavioral phenotype of HD. However, one study reported no changes in number of OXT neurons within the PVN (van Wamelen et al., 2012). This discrepancy in results may stem from different quantification approaches used. In the study from Gabery et al. stereological quantification with the physical dissector principle was applied while this was not the case in the study by van Wamelen et al. (2012).

In several studies, OXT has been measured in both blood and cerebrospinal fluid (CSF) samples from individuals with HD. A recent study showed a significant 38% reduction in OXT CSF levels in individuals carrying the mutant *HTT* gene (Hellem et al., 2022). No changes in OXT plasma levels in HD patients have been found, highlighting that changes in OXT levels occur centrally and not in the periphery (Unti et al., 2018; Fisher et al., 2021; Hellem et al., 2022).

There are a number of different transgenic mouse models for HD (Pouladi et al., 2013). A decrease in OXT mRNA levels in the CNS of both the R6/2 mouse expressing a short fragment of mutant *HTT* as well as HD190QG expressing a longer fragment of mutant *HTT* gene was observed (Kotliarova et al., 2005). A decrease of OXT mRNA levels has also been reported in a viral vector model with overexpression of mutant HTT selectively in the hypothalamus (Hult et al., 2011). Furthermore, R6/2 mice displayed loss of OXT-expressing neurons, which was not found in two other HD animal models; the BACHD and the HD190Q transgenic HD mice (Kotliarova et al., 2005; Soylu-Kucharz et al., 2016; Henningsen et al., 2021). In the PVN of HD190QG mice, lower levels of OXT mRNA were associated with a high frequency of mutant HTT aggregates (Kotliarova et al., 2005). Furthermore, pretreatment with OXT before intracerebroventricular (icv) injection 3-nitropropionic acid (3-NP)-induced HD mouse model prevented the development of several changes including decreased levels of the OXT receptor, mGluR2 and glutathione as well as increased mGluR5 levels in the striatum, hippocampus, prefrontal cortex, and amygdala (Khodagholi et al., 2022). These results indicate that OXT might have a protective effect on these molecular changes in HD. However, the OXT neuronal population appear to be resistant to quinolinic acid induced toxicity (Henningsen et al., 2021), which is an excitotoxin that has been linked to a loss of medium spiny neurons in the striatum of HD (Beal et al., 1986, 1991; Ferrante et al., 1993). This suggests that the vulnerability of OXT expressing neurons in HD is not caused by excitotoxicity. The underlying mechanisms of OXT loss in HD are not known and need further study.

### Effects of oxytocin on social cognition and psychiatric features in Huntington’s disease

Oxytocin has previously been established to have an important role in social cognition. Clinical studies have revealed a link between OXT and social cognition in HD. In HD patients, the ability to process emotions such as disgust, fear, anger, sad, surprise, and happiness is reduced (Labuschagne et al., 2018). Interestingly, a higher baseline level of OXT in the plasma was associated with a better recognition of emotion facial expression in HD gene carriers at an early disease stage before any onset of motor signs and symptoms onset (premanifest HD) (Unti et al., 2018). Moreover, intranasal OXT treatment normalized the ability of premanifest patients to process disgust stimuli (Labuschagne et al., 2018). More recently, Hellem et al. (2022) reported that HD patients with social cognitive impairment had significantly lower OXT CSF levels, suggesting a correlation between OXT CSF levels and social cognitive function. OXT may also be associated with executive dysfunction in HD. A clinical pilot study revealed that premanifest HD patients with higher OXT plasma levels performed better at cognitive tasks including verbal functioning, visual spatial attention, processing speed and working memory (Fisher et al., 2021). With an unmet clinical need for HD biomarkers, both OXT CSF and plasma levels may give some indication of the status of social cognitive deficits in HD.

Oxytocin could also play a role for the neuropsychiatric features of HD. In clinical HD, a positive correlation between OXT plasma levels and depression in both motor manifest and premanifest HD patients has been observed (Fisher et al., 2021). In the BACHD mouse model, OXT plasma level is lower than in wild-type littermates with increased depressive-, anxiety-like and social behavior (Cheong et al., 2020). Moreover, acute intranasal OXT administration reduced depressive-like behavior in this mouse model with no effect on anxiety-like behavior (Cheong et al., 2020). Furthermore, pretreatment with OXT injections prior to icv NP-3 injection in rats prevented the development of anxiety-and depressive-like behavior (Khodagholi et al., 2022). These results suggest that OXT might have protective effects and should be further investigated as a potential treatment in preventing the development of depressive and/or anxious phenotype in HD.

### Clinical features and neuropathology of frontotemporal dementia

Frontotemporal dementia is a group of NDDs that are characterized by progressive altered behavior and decline in executive functions. FTD is the second most common dementia after Alzheimer’s disease (Ahmed et al., 2021b). Clinically it is subdivided into three main types including behavioral-variant frontotemporal dementia (bv-FTD), semantic dementia and progressive non-fluent aphasia (Neary et al., 1998; Hodges and Patterson, 2007). bv-FTD is the most common form and comprises over 50% of all FTD cases. The syndrome has an early age of onset with a mean around 50 years of age and disease duration of approximately 8 years. Affected individuals have a range of clinical symptoms such as behavioral disinhibition, hallucinations, apathy, executive dysfunction as well as changes in eating behavior with hyperorality (Woolley et al., 2007; Rascovsky et al., 2011). However, a central feature in this condition is the early progressive loss of empathy and social cognition (Rankin et al., 2005). As such, considerable amount of research has during the past two decades been devoted to this topic. Impaired recognition of the facial expression of emotions occurs at an early stage, which in turn complicates the engagement or response to social cues (Keane et al., 2002; Rosen et al., 2004). Regions in the brain that are thought to be involved in social cognition include frontal, temporal and parietal lobes, which are the regions with predisposition to neuropathological changes and atrophy in FTD (Kennedy and Adolphs, 2012; Ahmed et al., 2021b).

Psychiatric symptoms can often initially mask an FTD diagnosis, as several of the symptoms overlap with other psychiatric syndromes such as obsessive-compulsive disorder, bipolar disorder and major depressive disorder (MDD). Around 50% of patients with bv-FTD receive initially a psychiatric diagnosis (Woolley et al., 2011). Neuroimaging studies have revealed a loss of gray matter in both MDD and bipolar disease that overlap with the affected regions in FTD (Peet et al., 2021). This, therefore, poses a diagnostic difficulty in the clinical setting (Ducharme et al., 2020). Recently, post-mortem analysis on brain tissue from FTD cases have shown a correlation between psychiatric symptoms and a higher abundance of the transactive response DNA-binding protein 43 kDa (TDP-43) inclusion pathology (Scarioni et al., 2020).

### Clinical features and neuropathology of amyotrophic lateral sclerosis

Amyotrophic lateral sclerosis is a fast-progressing NDD associated with both upper and lower motor neuron dysfunction leading to muscle weakness and bulbar dysfunction (Taylor et al., 2016). The disease onset occurs commonly in mid-adulthood (at a mean age of 55 years) with death typically 3–5 years after diagnosis usually due to respiratory failure (Zarei et al., 2015; Taylor et al., 2016). ALS patients also exhibit a range of non-motor symptoms including altered energy metabolism and eating behavior (Dupuis et al., 2011; Ahmed et al., 2016, 2021b). Pathological findings have been observed in the frontal and temporal cortices as well as in the hypothalamus that may underlie some of these changes (Neumann et al., 2006; Gabery et al., 2021).

Psychiatric symptoms such as apathy and depression have also been described in ALS, which have been shown to precede the onset of motor symptoms (Mioshi et al., 2014; Caga et al., 2018, 2021). In particular, depression is reported before and after diagnosis (Turner et al., 2016). Recent studies indicate that social cognitive impairment and emotion facial expression processing deficits is present in ALS patients. Also, an increased atrophy of the fornix, the main white matter tract of the limbic system, has shown to correlate with increased behavioral changes in ALS patients (Gabery et al., 2021). A recent report has shown that ALS patients have more difficulty with recognition of facial expression of emotions such as disgust, anger, fear and sadness (Palumbo et al., 2022). These findings suggest that the OXT pathway might be affected in ALS.

### Overlap between amyotrophic lateral sclerosis and frontotemporal dementia

In recent years, a growing amount of evidence point toward that ALS and a large proportion of FTD are part of a disease spectrum continuum (Clark and Forman, 2006). Approximately 15% of FTD patients develop ALS-associated motor signs and symptoms and 15–18% of ALS patients exhibit FTD-like symptoms (Burrell et al., 2011; Lattante et al., 2015; Taylor et al., 2016). This concept was potentiated further with the discovery of the C9ORF72 expansion causing both ALS and FTD (DeJesus-Hernandez et al., 2011; Hodges, 2012). Both conditions share overlap at the neuropathological level including cytoplasmic TDP-43 inclusions in both neurons and glia cells (Buratti and Baralle, 2008, 2012).

### Changes in the oxytocin system in amyotrophic lateral sclerosis and frontotemporal dementia

For FTD and ALS, the symptomatology and the clinical presentation suggest involvement of key physiological functions of the hypothalamus, which might even precede the onset of the cognitive and motor symptoms development (Vercruysse et al., 2018; Ahmed et al., 2021a). Significant atrophy of the hypothalamus is present on structural magnetic resonance imaging (MRI) as well as on post-mortem analyses in patients with bv-FTD and ALS (Piguet et al., 2011; Gorges et al., 2017; Gabery et al., 2021). So far, only a few studies have investigated OXT in ALS and FTD.

Recently, a 33% loss of OXT-expressing neurons in post-mortem tissue from ALS patients was reported together with the presence of TDP-43 inclusions in OXT-expressing neurons (Gabery et al., 2021). In bv-FTD, promising results have been observed during pharmacological treatments with OXT mainly targeting loss of empathy and the ability to process facial expression of emotions. So far, two small randomized controlled trials with intranasal OXT treatment have shown safety and tolerability as well as significant improvement in neuropsychiatric inventory scores assessing agitation, depression, apathy and irritability (Table 2; Jesso et al., 2011; Finger et al., 2015). However, larger randomized control trials are required before more definitive conclusions of the potential positive therapeutic effects of OXT can be made.

**TABLE 2:** Summary of oxytocin (OXT) results in amyotrophic lateral sclerosis (ALS) and frontotemporal dementia (FTD).

Tissue	Condition	Analysis	Treatment		Results	References
Clinical ALS/FTD	ALS	IHC, stereology		↓	Number of OXT neurons	Gabery et al., 2021
	FTD		Intranasal OXT adm.		Safe and well tolerated	Finger et al., 2015
	FTD	Facial expression recognition task, neuropsychiatric inventory scale	Intranasal OXT adm.	↑	Emotion recognition Improvement in neuropsychiatric inventory score	Jesso et al., 2011
	FTD	fMRI, Bold	Intranasal OXT adm.	↑	Increased activity in limbic regions	Oliver et al., 2020

↓: decrease in neuronal population, ↑: improvement in capacity. IHC, immunohistochemistry; fMRI, functional magnetic resonance imaging; BOLD, blood oxygenation level dependent.

Furthermore, using blood oxygen level dependent signal during functional MRI, intranasal OXT treatment showed enhanced activity in limbic regions associated with processing of facial expression of emotions (Oliver et al., 2020). However, to date, no studies have investigated OXT in animal models of ALS or FTD. Nevertheless, these findings together highlight the potential of OXT as symptomatic treatment for deficits.

### Similarities across Huntington’s disease and the spectrum of amyotrophic lateral sclerosis and frontotemporal dementia disorders

**FIGURE 1:**
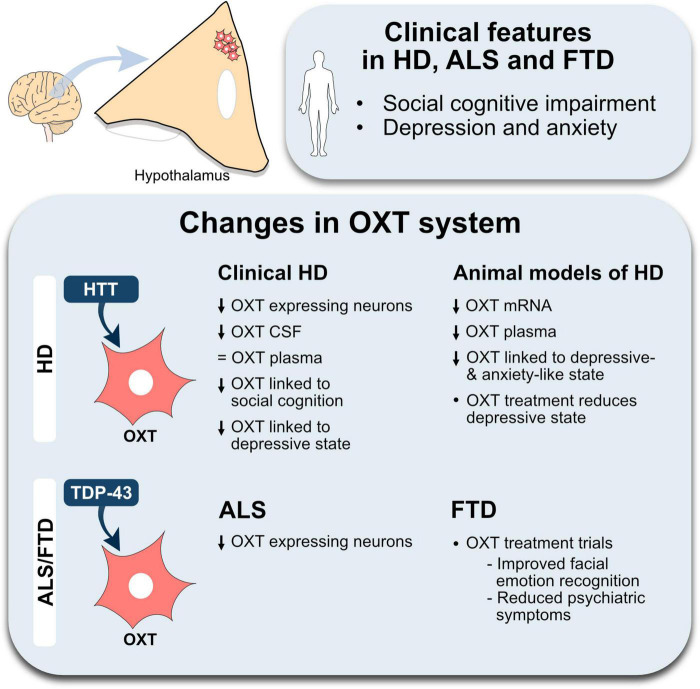
An overview of changes in the oxytocin system in Huntington’s disease and amyotrophic lateral sclerosis-frontotemporal dementia. The illustration summarizes the main positive results from studies investigating the oxytocin (OXT) system in Huntington’s disease (HD), amyotrophic lateral sclerosis (ALS) and frontotemporal dementia (FTD). A decrease is indicated by the downward arrows, lack of change is indicated by an equal sign. Abbreviations, CSF, cerebrospinal fluid; HTT, huntingtin; TDP-43, transactive response DNA-binding protein 43 kDa.

The role of OXT has evolved from solely being related to parturition and breastfeeding to be able to modulate aspects of social behavior as well as emotional regulation. As these functions are affected early on in HD and the spectrum of ALS/FTD, OXT may play an important role (Figure 1). Impairments in social cognition as well as a reduced ability to recognize facial expression of emotions in all three conditions have been established (Craufurd et al., 2001; Christidi et al., 2018; Keech et al., 2018; Palumbo et al., 2022). This could be associated with OXT, in particular in HD and FTD in which direct correlations of OXT levels has been found (Jesso et al., 2011; Labuschagne et al., 2018; Unti et al., 2018; Hellem et al., 2022). Moreover, hypothalamic pathology has been identified in all three conditions with a selective OXT loss in HD and ALS. Histopathological findings such as HTT inclusions in HD as well as presence of TDP-43 inclusions in OXT-expressing neurons suggest a selective vulnerability of this neuronal population to the presence of mutant HTT and TDP-43 (Gabery et al., 2010, 2021). To date, no studies have investigated the number of OXT cells in FTD. Collectively, these findings could indicate a mechanistic overlap across all three NDDs.

Furthermore, pharmacological administration with exogenous OXT improved processing of facial expression of emotions in both HD and FTD, thus supporting the possibility of a therapeutic application of OXT in the future. However, chronic administration of OXT at least in rodents lead to anxiety, via alternative splicing of *Crfr2a* (Winter et al., 2021), which needs to be considered in the development of clinical applications.

## Conclusion

In this review, we provided a summary of the main results implicating changes in the OXT system related to HD, ALS and FTD in the literature. Across all three conditions, the impairments in social cognition and neuropsychiatric behavior occur early in the disease progression, prior to the onset of motor disturbances. OXT neuropathology may at least in part explain the development of these early features. There is an unmet need for biomarkers to track early disease progression in HD, ALS, and FTD. Levels of OXT in both CSF and plasma have been shown to track certain social cognitive features, rendering OXT a potential biomarker candidate. Also, initial pharmacological intervention with OXT shows promising results. However, more experimental studies are needed to further determine the causative role of OXT in the development of the social and psychiatric impairments in HD, ALS, and FTD.

## Author contributions

All authors listed have made a substantial, direct, and intellectual contribution to the work, and approved it for publication.

## References

[B1] Ahmed R. M. Caga J. Devenney E. Hsieh S. Bartley L. Highton-Williamson E. (2016). Cognition and eating behavior in amyotrophic lateral sclerosis: Effect on survival. *J. Neurol.* 263 1593–1603. 10.1007/s00415-016-8168-2 27260291

[B2] Ahmed R. M. Steyn F. Dupuis L. (2021c). Hypothalamus and weight loss in amyotrophic lateral sclerosis. *Handb. Clin. Neurol.* 180 327–338. 10.1016/B978-0-12-820107-7.00020-3 34225938

[B3] Ahmed R. M. Hodges J. R. Piguet O. (2021b). Behavioural Variant Frontotemporal Dementia: Recent Advances in the Diagnosis and Understanding of the Disorder. *Adv. Exp. Med. Biol.* 1281 1–15. 10.1007/978-3-030-51140-1_1 33433865

[B4] Ahmed R. M. Halliday G. Hodges J. R. (2021a). Hypothalamic symptoms of frontotemporal dementia disorders. *Handb. Clin. Neurol.* 182 269–280. 10.1016/B978-0-12-819973-2.00019-8 34266598

[B5] Ahmed R. M. Macmillan M. Bartley L. Halliday G. M. Kiernan M. C. Hodges J. R. (2014). Systemic metabolism in frontotemporal dementia. *Neurology* 83 1812–1818. 10.1212/WNL.0000000000000993 25305153

[B6] Beal M. F. Kowall N. W. Ellison D. W. Mazurek M. F. Swartz K. J. Martin J. B. (1986). Replication of the neurochemical characteristics of Huntington’s disease by quinolinic acid. *Nature* 321 168–171. 10.1038/321168a0 2422561

[B7] Beal M. Ferrante R. Swartz K. Kowall N. (1991). Chronic quinolinic acid lesions in rats closely resemble Huntington’s disease. *J. Neurosci.* 11 1649–1659. 10.1523/JNEUROSCI.11-06-01649.1991 1710657 PMC6575424

[B8] Blasco H. Lanznaster D. Veyrat-Durebex C. Hergesheimer R. Vourch P. Maillot F. (2020). Understanding and managing metabolic dysfunction in Amyotrophic Lateral Sclerosis. *Expert Rev. Neurother.* 20 907–919. 10.1080/14737175.2020.1788389 32583696

[B9] Boentert M. (2020). Sleep and Sleep Disruption in Amyotrophic Lateral Sclerosis. *Curr. Neurol. Neurosci. Rep.* 20:25. 10.1007/s11910-020-01047-1 32462331 PMC7253511

[B10] Bott N. T. Radke A. Stephens M. L. Kramer J. H. (2014). Frontotemporal dementia: Diagnosis, deficits and management. *Neurodegener. Dis. Manag.* 4 439–454. 10.2217/nmt.14.34 25531687 PMC4824317

[B11] Buratti E. Baralle F. E. (2008). Multiple roles of Tdp-43 in gene expression, splicing regulation, and human disease. *Front. Biosci.* 13 867–878. 10.2741/2727 17981595

[B12] Buratti E. Baralle F. E. (2012). Tdp-43: Gumming up neurons through protein-protein and protein-Rna interactions. *Trends Biochem. Sci.* 37 237–247. 10.1016/j.tibs.2012.03.003 22534659

[B13] Burrell J. R. Kiernan M. C. Vucic S. Hodges J. R. (2011). Motor neuron dysfunction in frontotemporal dementia. *Brain* 134 2582–2594. 10.1093/brain/awr195 21840887

[B14] Caga J. Hsieh S. Highton-Williamson E. Zoing M. C. Ramsey E. Devenney E. (2018). Apathy and its impact on patient outcome in amyotrophic lateral sclerosis. *J. Neurol.* 265 187–193. 10.1007/s00415-017-8688-4 29189922

[B15] Caga J. Tu S. Dharmadasa T. Tse N. Y. Zoing M. C. Huynh W. (2021). Apathy is associated with parietal cortical-subcortical dysfunction in Als. *Cortex* 145 341–349. 10.1016/j.cortex.2021.02.029 33867121

[B16] Cheong R. Y. Gabery S. Petersén Å. (2019). The Role of Hypothalamic Pathology for Non-Motor Features of Huntington’s Disease. *J. Huntingtons Dis.* 8 375–391. 10.3233/JHD-190372 31594240 PMC6839491

[B17] Cheong R. Y. Tonetto S. Von Hörsten S. Petersén Å. (2020). Imbalance of the oxytocin-vasopressin system contributes to the neuropsychiatric phenotype in the Bachd mouse model of Huntington disease. *Psychoneuroendocrinology* 119:104773. 10.1016/j.psyneuen.2020.104773 32590293

[B18] Christidi F. Migliaccio R. Santamaría-García H. Santangelo G. Trojsi F. (2018). Social Cognition Dysfunctions in Neurodegenerative Diseases: Neuroanatomical Correlates and Clinical Implications. *Behav. Neurol.* 2018:1849794. 10.1155/2018/1849794 29854017 PMC5944290

[B19] Cisbani G. Cicchetti F. (2012). An in vitro perspective on the molecular mechanisms underlying mutant huntingtin protein toxicity. *Cell Death Dis.* 3 e382–e382. 10.1038/cddis.2012.121 22932724 PMC3434668

[B20] Clark C. M. Forman M. S. (2006). Frontotemporal lobar degeneration with motor neuron disease: A clinical and pathological spectrum. *Arch. Neurol.* 63 489–490. 10.1001/archneur.63.4.489 16606759

[B21] Craufurd D. Thompson J. C. Snowden J. S. (2001). Behavioral Changes in Huntington Disease. *Neuropsychiatry Neuropsychol. Behav. Neurol.* 14:219–26. 11725215

[B22] DeJesus-Hernandez M. Mackenzie I. R. Boeve B. F. Boxer A. L. Baker M. Rutherford N. J. (2011). Expanded Ggggcc hexanucleotide repeat in noncoding region of C9orf72 causes chromosome 9p-linked Ftd and Als. *Neuron* 72 245–256. 10.1016/j.neuron.2011.09.011 21944778 PMC3202986

[B23] DiFiglia M. Sapp E. Chase K. O. Davies S. W. Bates G. P. Vonsattel J. P. (1997). Aggregation of huntingtin in neuronal intranuclear inclusions and dystrophic neurites in brain. *Science* 277 1990–1993. 10.1126/science.277.5334.1990 9302293

[B24] Ducharme S. Dols A. Laforce R. Devenney E. Kumfor F. Van Den Stock J. (2020). Recommendations to distinguish behavioural variant frontotemporal dementia from psychiatric disorders. *Brain* 143 1632–1650. 10.1093/brain/awaa018 32129844 PMC7849953

[B25] Duff K. Paulsen J. S. Beglinger L. J. Langbehn D. R. Stout J. C. (2007). Psychiatric symptoms in Huntington’s disease before diagnosis: The predict-Hd study. *Biol. Psychiatry* 62 1341–1346. 10.1016/j.biopsych.2006.11.034 17481592

[B26] Dupuis L. Pradat P. F. Ludolph A. C. Loeffler J. P. (2011). Energy metabolism in amyotrophic lateral sclerosis. *Lancet Neurol.* 10 75–82. 10.1016/S1474-4422(10)70224-6 21035400

[B27] Duyao M. Ambrose C. Myers R. Novelletto A. Persichetti F. Frontali M. (1993). Trinucleotide repeat length instability and age of onset in Huntington’s disease. *Nat. Genet.* 4 387–392. 10.1038/ng0893-387 8401587

[B28] Elden A. C. Kim H. J. Hart M. P. Chen-Plotkin A. S. Johnson B. S. Fang X. (2010). Ataxin-2 intermediate-length polyglutamine expansions are associated with increased risk for Als. *Nature* 466 1069–1075. 10.1038/nature09320 20740007 PMC2965417

[B29] Ferrante R. J. Kowall N. W. Cipolloni P. B. Storey E. Beal M. F. (1993). Excitotoxin Lesions in Primates as a Model for Huntington’s Disease: Histopathologic and Neurochemical Characterization. *Exp. Neurol.* 119 46–71. 10.1006/exnr.1993.1006 8432351

[B30] Finger E. C. Mackinley J. Blair M. Oliver L. D. Jesso S. Tartaglia M. C. (2015). Oxytocin for frontotemporal dementia: A randomized dose-finding study of safety and tolerability. *Neurology* 84 174–181. 10.1212/WNL.0000000000001133 25503617 PMC4336088

[B31] Fisher E. R. Rocha N. P. Morales-Scheihing D. A. Venna V. R. Furr-Stimming E. E. Teixeira A. L. (2021). The Relationship Between Plasma Oxytocin and Executive Functioning in Huntington’s Disease: A Pilot Study. *J. Huntingtons Dis.* 10 349–354. 10.3233/JHD-210467 34092650

[B32] Fronczek R. Overeem S. Lee S. Y. Hegeman I. M. Van Pelt J. Van Duinen S. G. (2007). Hypocretin (orexin) loss in Parkinson’s disease. *Brain* 130 1577–1585. 10.1093/brain/awm090 17470494

[B33] Gabery S. Ahmed R. M. Caga J. Kiernan M. C. Halliday G. M. Petersén Å (2021). Loss of the metabolism and sleep regulating neuronal populations expressing orexin and oxytocin in the hypothalamus in amyotrophic lateral sclerosis. *Neuropathol. Appl. Neurobiol.* 47 979–989. 10.1111/nan.12709 33755993

[B34] Gabery S. Halliday G. Kirik D. Englund E. Petersén Å (2015). Selective loss of oxytocin and vasopressin in the hypothalamus in early Huntington disease: A case study. *Neuropathol. Appl. Neurobiol.* 41 843–848. 10.1111/nan.12236 25765897

[B35] Gabery S. Murphy K. Schultz K. Loy C. T. Mccusker E. Kirik D. (2010). Changes in key hypothalamic neuropeptide populations in Huntington disease revealed by neuropathological analyses. *Acta Neuropathol.* 120 777–788. 10.1007/s00401-010-0742-6 20821223

[B36] Galbally M. Lewis A. J. Ijzendoorn M. Permezel M. (2011). The role of oxytocin in mother-infant relations: A systematic review of human studies. *Harv. Rev. Psychiatry* 19 1–14. 10.3109/10673229.2011.549771 21250892

[B37] Gimpl G. Fahrenholz F. (2001). The oxytocin receptor system: Structure, function, and regulation. *Physiol. Rev.* 81 629–683. 10.1152/physrev.2001.81.2.629 11274341

[B38] Gorges M. Vercruysse P. Müller H. P. Huppertz H. J. Rosenbohm A. Nagel G. (2017). Hypothalamic atrophy is related to body mass index and age at onset in amyotrophic lateral sclerosis. *J. Neurol. Neurosurg. Psychiatry* 88 1033–1041. 10.1136/jnnp-2017-315795 28596251

[B39] Greaves C. V. Rohrer J. D. (2019). An update on genetic frontotemporal dementia. *J. Neurol.* 266 2075–2086. 10.1007/s00415-019-09363-4 31119452 PMC6647117

[B40] HDCRG (1993). A novel gene containing a trinucleotide repeat that is expanded and unstable on Huntington’s disease chromosomes The Huntington’s Disease Collaborative Research Group. *Cell* 72 971–983. 10.1016/0092-8674(93)90585-E 8458085

[B41] Heinrichs M. Von Dawans B. Domes G. (2009). Oxytocin, vasopressin, and human social behavior. *Front. Neuroendocrinol.* 30 548–557. 10.1016/j.yfrne.2009.05.005 19505497

[B42] Hellem M. N. N. Cheong R. Y. Tonetto S. Vinther-Jensen T. Hendel R. K. Larsen I. U. (2022). Decreased Csf oxytocin relates to measures of social cognitive impairment in Huntington’s disease patients. *Parkinsonism Relat. Disord.* 99 23–29. 10.1016/j.parkreldis.2022.05.003 35580426

[B43] Henningsen J. B. Soylu-Kucharz R. Björkqvist M. Petersén Å (2021). Effects of excitotoxicity in the hypothalamus in transgenic mouse models of Huntington disease. *Heliyon* 7:e07808. 10.1016/j.heliyon.2021.e07808 34458633 PMC8379469

[B44] Herzog-Krzywoszanska R. Krzywoszanski L. (2019). Sleep Disorders in Huntington’s Disease. *Front. Psychiatry* 10:221. 10.3389/fpsyt.2019.00221 31031659 PMC6474183

[B45] Hodges J. (2012). Familial frontotemporal dementia and amyotrophic lateral sclerosis associated with the C9orf72 hexanucleotide repeat. *Brain* 135 652–655. 10.1093/brain/aws033 22366789

[B46] Hodges J. R. Patterson K. (2007). Semantic dementia: A unique clinicopathological syndrome. *Lancet Neurol.* 6 1004–1014. 10.1016/S1474-4422(07)70266-1 17945154

[B47] Hult S. Soylu R. Björklund T. Belgardt Bengt F. Mauer J. Brüning Jens C (2011). Mutant Huntingtin Causes Metabolic Imbalance by Disruption of Hypothalamic Neurocircuits. *Cell Metab.* 13 428–439. 10.1016/j.cmet.2011.02.013 21459327

[B48] Jesso S. Morlog D. Ross S. Pell M. D. Pasternak S. H. Mitchell D. G. (2011). The effects of oxytocin on social cognition and behaviour in frontotemporal dementia. *Brain* 134 2493–2501. 10.1093/brain/awr171 21859765

[B49] Jurek B. Neumann I. D. (2018). The Oxytocin Receptor: From Intracellular Signaling to Behavior. *Physiol. Rev.* 98 1805–1908. 10.1152/physrev.00031.2017 29897293

[B50] Keane J. Calder A. J. Hodges J. R. Young A. W. (2002). Face and emotion processing in frontal variant frontotemporal dementia. *Neuropsychologia* 40 655–665. 10.1016/S0028-3932(01)00156-7 11792405

[B51] Keech B. Crowe S. Hocking D. R. (2018). Intranasal oxytocin, social cognition and neurodevelopmental disorders: A meta-analysis. *Psychoneuroendocrinology* 87 9–19. 10.1016/j.psyneuen.2017.09.022 29032324

[B52] Kennedy D. P. Adolphs R. (2012). The social brain in psychiatric and neurological disorders. *Trends Cogn. Sci.* 16 559–572. 10.1016/j.tics.2012.09.006 23047070 PMC3606817

[B53] Khodagholi F. Maleki A. Motamedi F. Mousavi M. A. Rafiei S. Moslemi M. (2022). Oxytocin Prevents the Development of 3-Np-Induced Anxiety and Depression in Male and Female Rats: Possible Interaction of Oxtr and mGluR2. *Cell. Mol. Neurobiol.* 42 1105–1123. 10.1007/s10571-020-01003-0 33201416 PMC11441245

[B54] Kotliarova S. Jana N. R. Sakamoto N. Kurosawa M. Miyazaki H. Nekooki M. (2005). Decreased expression of hypothalamic neuropeptides in Huntington disease transgenic mice with expanded polyglutamine-Egfp fluorescent aggregates. *J. Neurochem.* 93 641–653. 10.1111/j.1471-4159.2005.03035.x 15836623

[B55] Labuschagne I. Poudel G. Kordsachia C. Wu Q. Thomson H. Georgiou-Karistianis N. (2018). Oxytocin selectively modulates brain processing of disgust in Huntington’s disease gene carriers. *Prog. Neuropsychopharmacol. Biol. Psychiatry* 81 11–16. 10.1016/j.pnpbp.2017.09.023 28947180

[B56] Lattante S. Ciura S. Rouleau G. A. Kabashi E. (2015). Defining the genetic connection linking amyotrophic lateral sclerosis (Als) with frontotemporal dementia (Ftd). *Trends Genet.* 31 263–273. 10.1016/j.tig.2015.03.005 25869998

[B57] McCarter S. J. St Louis E. K. Boeve B. F. (2016). Sleep Disturbances in Frontotemporal Dementia. *Curr. Neurol. Neurosci. Rep.* 16:85. 10.1007/s11910-016-0680-3 27485946

[B58] Mioshi E. Caga J. Lillo P. Hsieh S. Ramsey E. Devenney E. (2014). Neuropsychiatric changes precede classic motor symptoms in Als and do not affect survival. *Neurology* 82 149–155. 10.1212/WNL.0000000000000023 24336140

[B59] Neary D. Snowden J. S. Gustafson L. Passant U. Stuss D. Black S. (1998). Frontotemporal lobar degeneration: A consensus on clinical diagnostic criteria. *Neurology* 51 1546–1554. 10.1212/WNL.51.6.1546 9855500

[B60] Neumann I. D. Landgraf R. (2012). Balance of brain oxytocin and vasopressin: Implications for anxiety, depression, and social behaviors. *Trends Neurosci.* 35 649–659. 10.1016/j.tins.2012.08.004 22974560

[B61] Neumann M. Sampathu D. M. Kwong L. K. Truax A. C. Micsenyi M. C. Chou T. T. (2006). Ubiquitinated Tdp-43 in frontotemporal lobar degeneration and amyotrophic lateral sclerosis. *Science* 314 130–133. 10.1126/science.1134108 17023659

[B62] Novak M. J. U. Tabrizi S. J. (2010). Huntington’s disease. *BMJ* 340:c3109. 10.1136/bmj.c3109 20591965

[B63] Odent M. R. (2013). Synthetic oxytocin and breastfeeding: Reasons for testing an hypothesis. *Med. Hypotheses* 81 889–891. 10.1016/j.mehy.2013.07.044 23948601

[B64] Oliver L. D. Stewart C. Coleman K. Kryklywy J. H. Bartha R. Mitchell D. G. V. (2020). Neural effects of oxytocin and mimicry in frontotemporal dementia: A randomized crossover study. *Neurology* 95 e2635–e2647. 10.1212/WNL.0000000000010933 32963103 PMC7713736

[B65] Onaka T. Takayanagi Y. (2019). Role of oxytocin in the control of stress and food intake. *J. Neuroendocrinol.* 31:e12700. 10.1111/jne.12700 30786104 PMC7217012

[B66] Palumbo F. Iazzolino B. Peotta L. Canosa A. Manera U. Grassano M. (2022). Social cognition deficits in amyotrophic lateral sclerosis: A pilot cross-sectional population-based study. *Eur. J. Neurol.* 29:2211–2219 10.1111/ene.15388 35524505 PMC9541579

[B67] Paulsen J. S. Langbehn D. R. Stout J. C. Aylward E. Ross C. A. Nance M. (2008). Detection of Huntington’s disease decades before diagnosis: The Predict-Hd study. *J. Neurol. Neurosurg. Psychiatry* 79 874–880. 10.1136/jnnp.2007.128728 18096682 PMC2569211

[B68] Peet B. T. Castro-Suarez S. Miller B. L. (2021). The Neuropsychiatric Features of Behavioral Variant Frontotemporal Dementia. *Adv. Exp. Med. Biol.* 1281 17–31. 10.1007/978-3-030-51140-1_2 33433866

[B69] Phukan J. Pender N. P. Hardiman O. (2007). Cognitive impairment in amyotrophic lateral sclerosis. *Lancet Neurol.* 6 994–1003. 10.1016/S1474-4422(07)70265-X 17945153

[B70] Piguet O. Petersén A. Yin, Ka Lam B. Gabery S. Murphy K. (2011). Eating and hypothalamus changes in behavioral-variant frontotemporal dementia. *Ann. Neurol.* 69 312–319. 10.1002/ana.22244 21387376 PMC3084499

[B71] Politis M. Pavese N. Tai Y. F. Tabrizi S. J. Barker R. A. Piccini P. (2008). Hypothalamic involvement in Huntington’s disease: An in vivo Pet study. *Brain* 131 2860–2869. 10.1093/brain/awn244 18829696

[B72] Pouladi M. A. Morton A. J. Hayden M. R. (2013). Choosing an animal model for the study of Huntington’s disease. *Nat. Rev. Neurosci.* 14 708–721. 10.1038/nrn3570 24052178

[B73] Rankin K. P. Kramer J. H. Miller B. L. (2005). Patterns of cognitive and emotional empathy in frontotemporal lobar degeneration. *Cogn. Behav. Neurol.* 18 28–36. 10.1097/01.wnn.0000152225.05377.ab 15761274

[B74] Rascovsky K. Hodges J. R. Knopman D. Mendez M. F. Kramer J. H. Neuhaus J. (2011). Sensitivity of revised diagnostic criteria for the behavioural variant of frontotemporal dementia. *Brain* 134 2456–2477. 10.1093/brain/awr179 21810890 PMC3170532

[B75] Renton A. E. Majounie E. Waite A. Simón-Sánchez J. Rollinson S. Gibbs J. R. (2011). A hexanucleotide repeat expansion in C9orf72 is the cause of chromosome 9p21-linked Als-Ftd. *Neuron* 72 257–268. 10.1016/j.neuron.2011.09.010 21944779 PMC3200438

[B76] Rokicki J. Kaufmann T. De Lange A. G. Van Der Meer D. Bahrami S. Sartorius A. M. (2022). Oxytocin receptor expression patterns in the human brain across development. *Neuropsychopharmacology* 47 1550–1560. 10.1038/s41386-022-01305-5 35347267 PMC9205980

[B77] Rosen H. J. Pace-Savitsky K. Perry R. J. Kramer J. H. Miller B. L. Levenson R. W. (2004). Recognition of emotion in the frontal and temporal variants of frontotemporal dementia. *Dement. Geriatr. Cogn. Disord.* 17 277–281. 10.1159/000077154 15178936

[B78] Ross C. A. Shoulson I. (2009). Huntington disease: Pathogenesis, biomarkers, and approaches to experimental therapeutics. *Parkinsonism Relat. Disord.* 15 S135–S138. 10.1016/S1353-8020(09)70800-4 20082975

[B79] Ross C. A. Aylward E. H. Wild E. J. Langbehn D. R. Long J. D. Warner J. H. (2014). Huntington disease: Natural history, biomarkers and prospects for therapeutics. *Nat. Rev. Neurol.* 10 204–216. 10.1038/nrneurol.2014.24 24614516

[B80] Saiz-Rodríguez M. Gil-Polo C. Diez-Fairen M. Martinez-Horta S. I. Sampedro Santalo F. Calvo S. (2022). Polymorphisms in the oxytocin receptor and their association with apathy and impaired social cognition in Huntington’s disease. *Neurol. Sci.* [Epub ahead of print]. 10.1007/s10072-022-06226-1 35725858

[B81] Scarioni M. Gami-Patel P. Timar Y. Seelaar H. Van Swieten J. C. Rozemuller A. J. M. (2020). Frontotemporal Dementia: Correlations Between Psychiatric Symptoms and Pathology. *Ann. Neurol.* 87 950–961. 10.1002/ana.25739 32281118 PMC7318614

[B82] Singh A. Agrawal N. (2021). Metabolism in Huntington’s disease: A major contributor to pathology. *Metab. Brain Dis.* 37 1757–1771 10.1007/s11011-021-00844-y 34704220

[B83] Soneson C. Fontes M. Zhou Y. Denisov V. Paulsen J. S. Kirik D. (2010). Early changes in the hypothalamic region in prodromal Huntington disease revealed by Mri analysis. *Neurobiol. Dis.* 40 531–543. 10.1016/j.nbd.2010.07.013 20682340 PMC2955781

[B84] Soylu-Kucharz R. Baldo B. Petersén Å (2016). Metabolic and behavioral effects of mutant huntingtin deletion in Sim1 neurons in the Bachd mouse model of Huntington’s disease. *Sci. Rep.* 6:28322. 10.1038/srep28322 27334347 PMC4917832

[B85] Steenland K. Macneil J. Seals R. Levey A. (2010). Factors affecting survival of patients with neurodegenerative disease. *Neuroepidemiology* 35 28–35. 10.1159/000306055 20389122 PMC2919432

[B86] Strong M. J. Abrahams S. Goldstein L. H. Woolley S. Mclaughlin P. Snowden J. (2017). Amyotrophic lateral sclerosis - frontotemporal spectrum disorder (Als-Ftsd): Revised diagnostic criteria. *Amyotroph. Lateral. Scler. Frontotemporal. Degener.* 18 153–174. 10.1080/21678421.2016.1267768 28054827 PMC7409990

[B87] Taylor J. P. Brown R. H. Jr. Cleveland D. W. (2016). Decoding Als: From genes to mechanism. *Nature* 539 197–206. 10.1038/nature20413 27830784 PMC5585017

[B88] Turner M. R. Goldacre R. Talbot K. Goldacre M. J. (2016). Psychiatric disorders prior to amyotrophic lateral sclerosis. *Ann. Neurol.* 80 935–938. 10.1002/ana.24801 27761925 PMC5215396

[B89] Tyree S. M. Borniger J. C. De Lecea L. (2018). Hypocretin as a Hub for Arousal and Motivation. *Front. Neurol.* 9:413. 10.3389/fneur.2018.00413 29928253 PMC5997825

[B90] Unti E. Mazzucchi S. Frosini D. Pagni C. Tognoni G. Palego L. (2018). Social Cognition and Oxytocin in Huntington’s Disease: New Insights. *Brain Sci.* 8:161. 10.3390/brainsci8090161 30149684 PMC6162368

[B91] van Wamelen D. J. Aziz N. A. Anink J. J. Roos R. A. Swaab D. F. (2012). Paraventricular nucleus neuropeptide expression in Huntington’s disease patients. *Brain Pathol.* 22 654–661. 10.1111/j.1750-3639.2012.00565.x 22257050 PMC8057638

[B92] Vercruysse P. Vieau D. Blum D. Petersén Å Dupuis L. (2018). Hypothalamic Alterations in Neurodegenerative Diseases and Their Relation to Abnormal Energy Metabolism. *Front. Mol. Neurosci.* 11:2. 10.3389/fnmol.2018.00002 29403354 PMC5780436

[B93] Vonsattel J. P. Myers R. H. Stevens T. J. Ferrante R. J. Bird E. D. Richardson E. P. Jr. (1985). Neuropathological classification of Huntington’s disease. *J. Neuropathol. Exp. Neurol.* 44 559–577. 10.1097/00005072-198511000-00003 2932539

[B94] Winter J. Meyer M. Berger I. Royer M. Bianchi M. Kuffner K. (2021). Chronic oxytocin-driven alternative splicing of Crfr2α induces anxiety. *Mol. Psychiatry* [Epub ahead of print]. 10.1038/s41380-021-01141-x 34035479 PMC10914602

[B95] Woolley J. D. Gorno-Tempini M. L. Seeley W. W. Rankin K. Lee S. S. Matthews B. R. (2007). Binge eating is associated with right orbitofrontal-insular-striatal atrophy in frontotemporal dementia. *Neurology* 69 1424–1433. 10.1212/01.wnl.0000277461.06713.23 17909155

[B96] Woolley J. D. Khan B. K. Murthy N. K. Miller B. L. Rankin K. P. (2011). The diagnostic challenge of psychiatric symptoms in neurodegenerative disease: Rates of and risk factors for prior psychiatric diagnosis in patients with early neurodegenerative disease. *J. Clin. Psychiatry* 72 126–133. 10.4088/JCP.10m06382oli 21382304 PMC3076589

[B97] Yoon S. Kim Y. K. (2020). The Role of the Oxytocin System in Anxiety Disorders. *Adv. Exp. Med. Biol.* 1191 103–120. 10.1007/978-981-32-9705-0_7 32002925

[B98] Zarei S. Carr K. Reiley L. Diaz K. Guerra O. Altamirano P. F. (2015). A comprehensive review of amyotrophic lateral sclerosis. *Surg. Neurol. Int.* 6:171. 10.4103/2152-7806.169561 26629397 PMC4653353

